# Engineered miR-122 inhibitors preserve endothelial mitochondrial function and prevent vascular dysfunction in obesity-associated prediabetes

**DOI:** 10.1016/j.omtn.2026.102830

**Published:** 2026-01-09

**Authors:** Ravinder Reddy Gaddam, Mounika Pathuri, Paroma Deb, Subhash Dwivedi, Anamika Vikram, Vishal Kasina, Veda S. Amalkar, Vitor Lira, Harpreet Kaur, Nirav Dhanesha, Ashutosh Kumar Mangalam, Raman Bahal, Ajit Vikram

**Affiliations:** 1 Division of Cardiovascular Medicine, Department of Internal Medicine, University of Iowa Carver College of Medicine, Iowa City, IA 52242, USA; 2 Abboud Cardiovascular Research Center, University of Iowa Carver College of Medicine, Iowa City, IA 52242, USA; 3 Fraternal Order of Eagles Diabetes Research Center (FOEDRC), University of Iowa Carver College of Medicine, Iowa City, IA 52242, USA; 4 Department of Pharmaceutical Sciences, University of Connecticut, Storrs, CT 06269, USA; 5 Department of Health, Sport, and Human Physiology, College of Liberal Arts and Sciences, University of Iowa, Iowa City, IA 52242, USA; 6 Department of Pathology and Translational Pathobiology, Louisiana State University Health Sciences Center-Shreveport, Shreveport, LA 71103, USA; 7 Department of Pathology, University of Iowa Carver College of Medicine, Iowa City, IA 52242, USA

**Keywords:** MT: Non-coding RNAs, miR-122, diabetes, endothelial dysfunction, miR inhibitors, neuropilin-1, mitochondrial function, oxidative phosphorylation, hyperglycemia

## Abstract

MicroRNA-122-5p (miR-122) is primarily expressed in the liver and is increasingly released into the bloodstream during obesity. It impacts the function of non-liver tissues, such as vascular endothelial cells, and increases the risk of diabetic vasculopathy. Using a gamma-peptide-nucleic acid-based miR-122 inhibitor (γP-122-I), we show that miR-122 regulates blood glucose levels and endothelial function in high-fat diet-fed mice. Targeting γP-122-I to endothelial cells retains its ability to improve vascular function but reduces metabolic benefits compared to the non-targeted version. Our results show that endothelial cells take up miR-122 through a neuropilin-1-dependent mechanism. Aortic transcriptomic analysis implicates miR-122 role in mitochondrial function. The aortas of high-fat diet-fed mice receiving an inhibitor of miR-122 were more efficient in oxygen consumption despite a decline in the expression of mitochondrial electron transport chain complexes. Supporting these findings, the overexpression of miR-122 under hyperglycemic conditions decreases mitochondrial electron transport chain respiration and mitochondria with high membrane potential, indicating its detrimental impact on mitochondrial function. These findings support miR-122 as a therapeutic target for diabetic vasculopathy and support γPNA-based miR-122 inhibition as a potentially safer and more effective therapy.

## Graphical abstract

**Figure:**
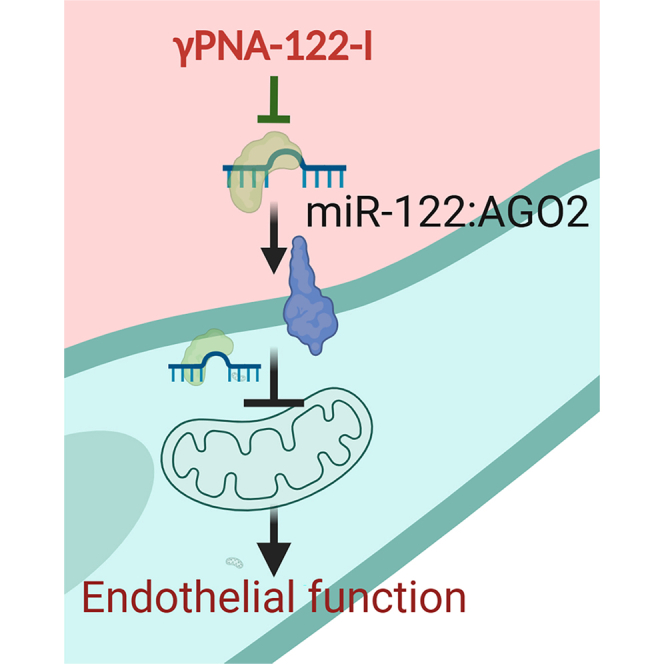

## Teaser

Endothelial uptake of microRNA-122 via neuropilin-1 disrupts mitochondrial respiration. γ-peptide nucleic acid-based inhibition of microRNA-122 preserves mitochondrial coupling efficiency and rescues endothelial vasorelaxation in obesity-associated prediabetes in mice.

## Introduction

MicroRNA-122-5p (miR-122) is predominantly expressed in hepatocytes and plays a crucial role in hepatic function. Recent studies have identified its presence and functional impact in non-hepatic tissues, including blood vessels and pancreatic islets.^1^^,^^2^ Certain health conditions, such as obesity, microbial dysbiosis, and diabetes, increase hepatic release of miR-122 into the circulation as a miR-122-Argonaute 2 (AGO2) complex.^3^^,^^4^^,^^5^ AGO2 is the primary component of the miR-induced silencing complex, and miR-122 occupies approximately 25% of AGO2 in hepatocytes.^3^ Thus, AGO2: miR-122 could functionally impact vascular endothelial cells without competing for the existing cellular AGO2 reserve. The impact of circulating miR-122 on glycemic control is significant, as evidenced by its role in breast cancer.^6^ Specifically, miR-122 is secreted by cancerous cells and decreases insulin release by pancreatic beta cells and glucose consumption by non-cancerous cells, all contributing to the increased availability of glucose to breast cancer cells.^6^ Notably, breast cancer survivors are at a higher risk of developing diabetes.^7^^,^^8^ miR-122 has been linked to endothelial cell apoptosis, endothelial-to-mesenchymal transition, and the development of atherosclerosis.^9^^,^^10^^,^^11^ miR-122 is a prominent regulator of miR-response elements in endothelial cells.^1^ Elevated blood levels of miR-122 contribute to diabetic vasculopathy.^12^^,^^13^^,^^14^ These observations suggest that targeting extrahepatic miR-122 could mitigate diabetic vasculopathy. Commercially available miR inhibitors bind strongly to proteins, have long half-lives, and are associated with nonspecific tissue accumulation and adverse outcomes.^15^^,^^16^^,^^17^ In clinical trials, miR-122 inhibitors have been tested as a treatment for hepatitis C but have faced setbacks due to immunoreactivity and hepatotoxicity. Modifying the chemical structure of miR-122 inhibitors could eliminate these side effects, thereby significantly improving their translational potential. We developed γ-peptide nucleic acid (γPNA)-based miR-122 inhibitors (γP-122-I), in which the phosphodiester backbone is replaced with an N-(2-aminoethyl) glycine backbone. γPNA is charge-neutral, water-soluble, and minimally binds to proteins.^18^ We recently reported that γP-122-I improves endothelial cell function and glycemic control in high-fat diet (HFD)-fed mice.^9^ This study begins to reveal potential mechanisms by investigating whether miR-122 affects mitochondrial function in vascular endothelial cells and whether endothelial cell-targeted γP-122-I (e-γP-122-I) offers a competitive advantage over non-targeted miR-122 inhibitors in mitigating endothelial dysfunction.

## Results

### Comparative analysis of systemic and endothelial cell-targeted miR-122 inhibition on metabolic and vascular parameters in HFD-fed mice

Our previous report demonstrates that systemic miR-122 inhibition using γP-122-I enhances endothelium-dependent vascular relaxation and glycemic control.^9^ We aimed to discern the metabolic and endothelial effects of miR-122 inhibition by comparing γP-122-I and e-γP-122-I to determine whether the latter offers advantages in efficacy and safety.

**Figure 1:**
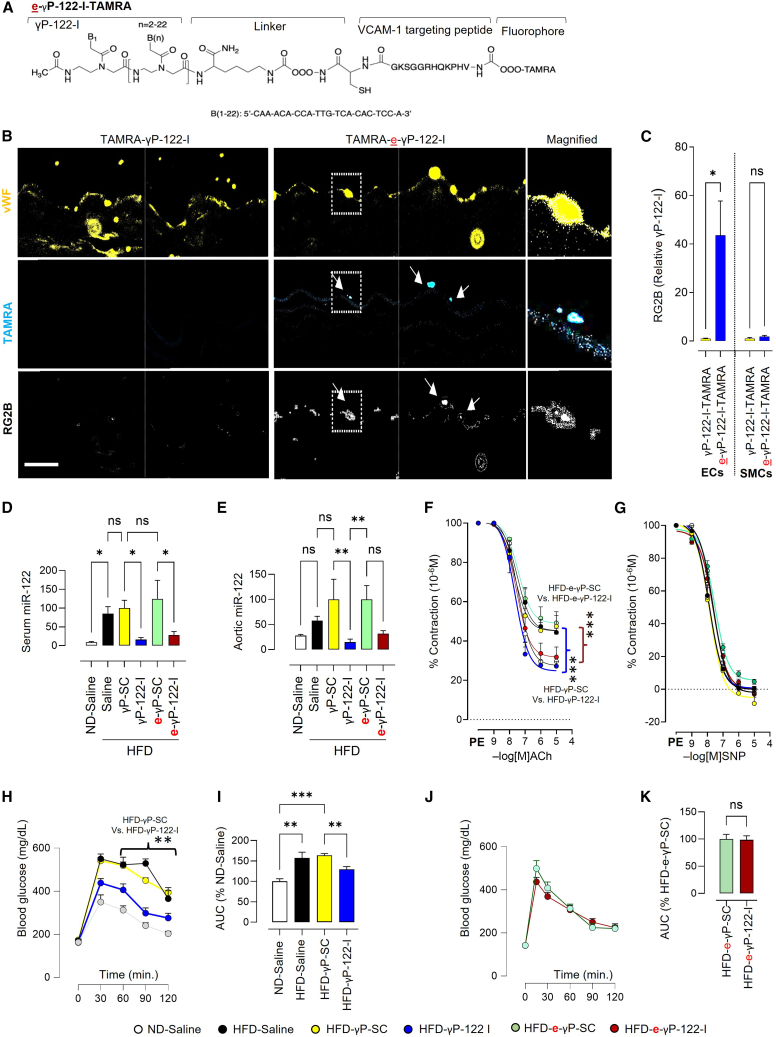
Vascular and metabolic effects of γP-122-I and e-γP-122-I (A) Schematic showing the design of TAMRA-e-γP-122-I. (B) Representative image showing immunostaining for the endothelial marker vWF (yellow) and fluorescence of TAMRA (cyan) in the aorta of mice receiving TAMRA-γP-122-I (non-targeted) or TAMRA-e-γP-122-I (endothelium targeted). Magnification ×63. Scale bars, 20 μm. (C) Quantification of miR-122 in the endothelial and smooth muscle (media) layers of the aorta treated with either TAMRA-γP-122-I or TAMRA-e-γP-122-I. The RG2B colocalization plugin in ImageJ was used to determine co-localization. *n* = 3. (D and E) miR-122 expression in the serum (D; *n* = 4–9) and aorta (E; *n* = 4–11) of normal diet-fed mice receiving saline (ND-saline) and HFD-fed mice receiving saline, γP-SC, γP-122-I, e-γP-SC, or e-γP-122-I. These mice received oligonucleotides at 0.25 μmol.kg^−1^ for 6 weeks. (F and G) γP-122-I and e-γP-122-I prevent HFD-triggered endothelial dysfunction in the aorta (F) but did not affect SNP-mediated relaxation (G; *n* = 5–8). (H–K) Blood glucose levels and time curves demonstrating the effects of γP-122-I (H and I; *n* = 5–19) and e-γP-122-I (J and K; *n* = 11) on glucose disposal during an intraperitoneal glucose tolerance test. The area under the curve (AUC) shows quantification of the glucose-time curve. ^ns^*p* > 0.05, ∗*p* < 0.05, ∗∗*p* < 0.01, and ∗∗∗*p* < 0.001 vs. the indicated group. Data are shown as mean ± S.E.M.

To generate e-γP-122-I, the vascular cell adhesion molecule-1 (VCAM-1)-targeting and internalizing peptide VHPK (*val-his-pro-lys-gln-his-arg-gln-gln-ser-lys-gln-cys*) was conjugated to γP-122-I. VCAM-1 is highly expressed in endothelial cells.^19^ Briefly, the PNAs/γPNAs oligomers were synthesized using standard Boc chemistry, purified by high-performance liquid chromatography (HPLC), and mass confirmed with matrix-assisted laser desorption/ionization-time of flight (MALDI-TOF) spectrometry (Figures S1A–S1C). A small quantity of e-γP-scramble control (e-γP-SC) and e-γP-122-I was conjugated with Tetramethylrhodamine (TAMRA) to facilitate visualization (Figure 1A). The colocalization of e-γP-122-I-TAMRA with the endothelial cell marker von Willebrand factor (vWF) in aortic sections confirmed endothelial targeting (Figures 1B and 1C). Mice receiving γP-122-I-TAMRA served as controls. Because the aortic transverse section provides a thin layer of endothelial cells, we also imaged the exposed endothelial surface of the aorta, further validating endothelial binding of e-γP-122-I (Figure S1D).

To test the impact of specific inhibition of miR-122 in the endothelium in a pre-clinical model of prediabetes, 8-week-old wild-type (C57BL/6J) mice were fed an HFD for 8 weeks and treated with γP-SC, γP-122-I, e-γP-SC, and e-γP-122-I at 0.25 μmol.kg^−1^ for the last 6 weeks of the intervention. Normal diet (ND)-fed mice were included as controls. Both γP-122-I and e-γP-122-I decreased blood and aortic miR-122 and mitigated HFD-triggered impairment in vascular endothelial function (Figures 1D–1F). Typically, γPNAs-miR complexes do not recruit RNase-H1 for cleavage; instead, they act as a sponge for miR and inhibit its function by steric hindrance on the target mRNA.^20^^,^^21^^,^^22^ In fact, we previously showed that γP-122-I forms a heteroduplex with miR-122 with very high affinity (high Tm).^9^ Therefore, the observed effects of miR-122 inhibition arise from functional suppression of the miR, not from its degradation. The γP-122-I had minimal effects on other miRs (e.g., miR-29b, miR-148a, miR-133a) in the aorta (Figures S2A–S2C). Compared to γP-122-I, e-γP-122-I had milder effects on miR-122 expression in the pancreas and kidney, while both exhibited similar effects in the liver (Figures S2D–S2F). Among the top predicted miR-122 target genes (*B2163fpl4, Cpeb1, Lama2, Tgfb1i1, Ddx60, Apobec4, Hivep3, Bnc2, Tnpo3*), we assessed the expression of *Cpeb1, Tgfb1i1, Bnc2, Ddx60, and Lama2* in the liver and kidney. In HFD-fed mice, γP-122-I tended to elicit greater induction of *Cpeb1, Tgfb1i1,* and *Bnc2* in the liver and *Cpeb1, Tgfb1i1, Ddx60,* and *Lama2* in the kidney, compared with e-γP-122-I (Figure S3). These trends align with the more restricted tissue distribution of the endothelial-targeted inhibitor. The absence of a difference between control and treatment groups in vascular relaxation by the endothelium-independent nitric oxide donor sodium nitroprusside (SNP) indicates that γP-122-I and e-γP-122-I work by improving nitric oxide-dependent vasorelaxation (Figure 1G).

To test efficacy in a more advanced diabetic state, we evaluated e-γP-122-I in *db/db* mice. As expected, the *db/db* mice were obese and hyperglycemic compared to the *db/+* mice (Figures S4A and S4B). The *db/+* and *db/db* mice receiving e-γP-SC served as controls for the *db/db* mice receiving e-γP-122-I. The e-γP-122-I significantly decreased aortic miR-122 levels and improved vascular endothelial function despite having no effect on body weight or random blood glucose levels (Figures S4A–S4E). The absence of a difference between control and treatment groups in vascular relaxation by SNP indicates that e-γP-122-I works by improving nitric oxide-dependent vasorelaxation (Figure S4E).

The γP-122-I and e-γP-122-I did not differ in their effects on body weight, adiposity, and cholesterol levels (Figures S5A–S5C). Consistent with our prior work, γP-122-I improved glucose tolerance (Figures 1H and 1I), whereas e-γP-122-I did not (Figures 1J and 1K), suggesting that systemic miR-122 inhibition exerts broader metabolic effects than endothelial-targeted delivery. We previously did not observe any signs of liver or kidney toxicity during 6 weeks of treatment with γP-122-I by histology.^9^ Similarly, we did not observe any signs of liver or kidney toxicity during 6 weeks of treatment with e-γP-122-I by histology (Figure S5D).

### miR-122 enters endothelial cells through NRP-1

**Figure 2:**
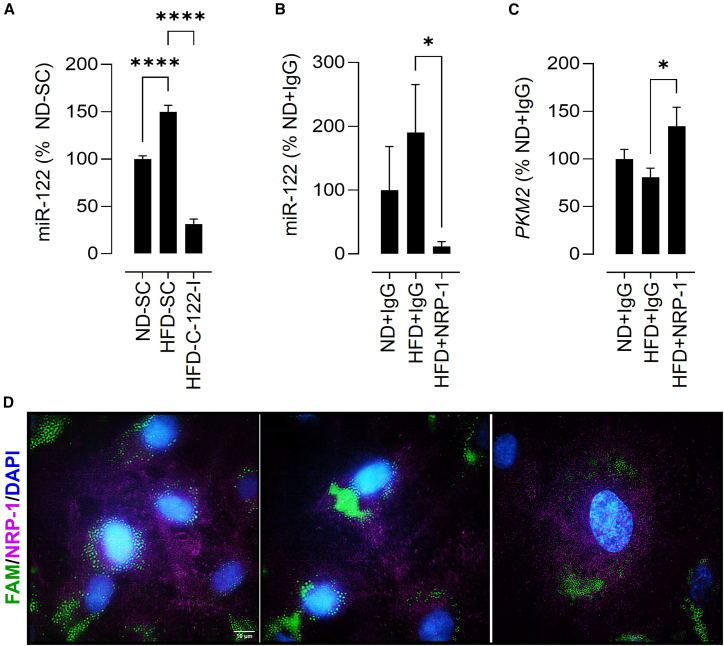
Endothelial cells uptake miR-122 via NRP-1 (A) miR-122 levels in HUVECs grown in media supplemented with 2% serum from ND-SC, HFD-SC, or HFD-C-122-I mice (*n* = 6–12). (B and C) Effect of an NRP-1 antibody on miR-122 uptake by HUVECs and on the expression of the miR-122 target gene PKM2 (*n* = 6–12). ND = normal diet; HFD = high-fat diet; SC = scrambled control. (D) Images showing miR-122-FAM and NRP-1 in endothelial cells grown in media supplemented with serum from HFD-fed mice receiving miR-122 FAM (×60). One-way ANOVA followed by Tukey’s test was used to compare groups. ∗*p* < 0.05, and ∗∗∗∗*p* < 0.001 vs. the indicated group. Data are shown as mean ± S.E.M.

Growing human umbilical vein endothelial cells (HUVECs) in media supplemented with mouse serum provides an *in vitro* method for determining the effects of serum factors.^23^ We found higher miR-122 levels in HUVECs cultured with serum from HFD-fed mice, but not when the serum came from HFD-fed mice treated with a miR-122 inhibitor (Figure 2A), suggesting that HUVECs take up miR-122 from the media. Neuropilin-1 (NRP-1) internalizes AGO2-linked miRs *via* the extracellular b1b2 domain, and NRP-1 is highly expressed in endothelial cells.^24^^,^^25^ The antibody that neutralizes the b1b2 domain of NRP-1 decreases miR-122 levels and increases the expression of the miR-122 target gene *PKM2* in HUVECs cultured with serum from HFD-fed mice (Figures 2B and 2C). Next, we immunostained HUVECs cultured with serum from HFD-fed mice treated with 6-Carboxyfluorescein (FAM)-labeled miR-122 (miR-122-FAM) for NRP-1. miR-122-FAM and NRP-1 were present in the endothelial cells (Figure 2D), supporting the notion that miR-122 is internalized by NRP-1-expressing endothelial cells.

### Inhibition of miR-122 improves mitochondrial function in the aorta

**Figure 3:**
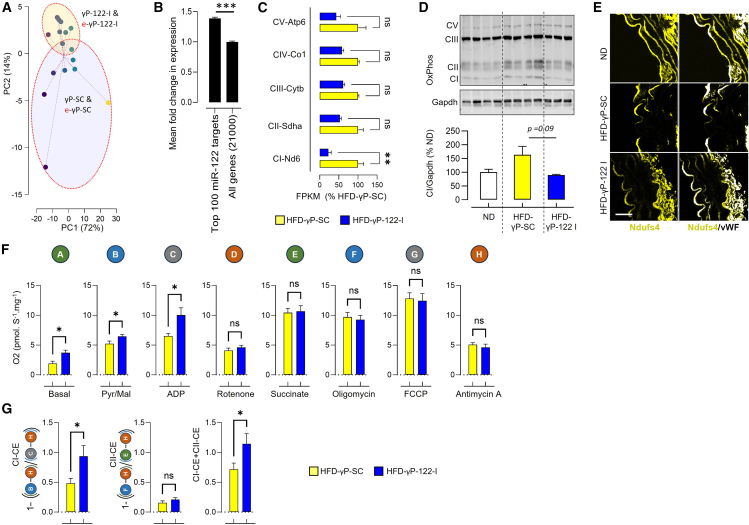
miR-122 regulates mitochondrial function in the vasculature (A) Principal component analysis (PCA) shows that the miR-122 inhibitor (γP-122-I) and the endothelium-targeted miR-122 inhibitor (e-γP-122-I) have similar effects on mouse aortic transcriptomics. The yellow distribution ellipse marks these samples. *n* = 3 + 5. Controls treated with SC (γP-SC) or endothelium-targeted SC (e-γP-SC) are marked by the violet distribution ellipse. *n* = 4 + 4. HFD-fed mice were treated with SC or a miR-122 inhibitor for 6 weeks (0.25 μmol.kg^−1^). The color of each individual sample represents its position along the PC1 axis. (B) Mean expression level of the top 100 upregulated miR-122 target genes compared with all other genes. (C) Expression of ETC complexes CI, CII, CIII, CIV, and CV in the aortic transcriptomics analysis *n* = 3–4. FPKM, fragments per kilobase of transcript per million mapped reads. (D) Immunoblot showing the expression of ETC complexes CI, CII, CIII, and CV in the aorta of HFD-fed mice receiving γP-122-I or γP-SC. Age-matched control mice fed a normal diet (ND) were also included. Bottom: quantification of CI in ND, HFD-γP-SC, and HFD-γP-122-I (*n* = 3–4). (E) Representative images showing the expression of Ndufs4, a subunit of complex I, in the aorta. vWF marks endothelial cells. (F) Oxygen (O_2_) consumption rate of permeabilized aorta under basal conditions and during sequential addition of pyruvate/malate (Pyr/Mal), ADP, rotenone, succinate, oligomycin, FCCP, and antimycin A (*n* = 4–6). (G) Coupling efficiency calculated by subtracting the ratio of leak-associated oxygen flux to oxphos-associated oxygen flux from 1 (*n* = 4–6). ∗*p* < 0.05, and ∗∗∗*p* < 0.01 *vs.* the indicated group. Data are shown as mean ± S.E.M.

We performed aortic transcriptomic analysis to determine whether and how miR-122 inhibition affects vascular function. In principle component analysis, the HFD-fed mice treated with either γP-122-I or e-γP-122-I clustered together, suggesting a similar pattern of change in gene expression (Figure 3A). Similarly, the control mice receiving non-targeted SC (γP-SC) or endothelium-targeted SC (e-γP-SC) clustered together (Figure 3A). Several miR-122 target genes were differently regulated in the aorta of mice receiving miR-122 inhibitors. The mean fold change of the top 100 upregulated miR-122 target genes was about 40% higher than the mean fold change of all other genes (Figure 3B). Ingenuity pathway analysis of the aortic transcriptomics of HFD-fed mice receiving either γP-SC or γP-122-I identified the role of miR-122 in cholesterol biosynthesis, lipid metabolism, xenobiotics handling, and molecular transport (Tables S1 and S2). Gene ontology enrichment analysis of the transcriptomics identified the role of miR-122 in biological processes such as B cell receptor signaling, lipid metabolism, and response to hormone; cellular compartments such as cytoplasm, cell membrane, cell surface, and endoplasmic reticulum; and molecular functions such as identical protein binding, oxidoreductase activity, and transmembrane transporter activity (Table S3). Mitochondria, the tricarboxylic acid cycle, and oxidative stress play a central role in cellular oxidoreductase activity. Therefore, to delve deeper, we performed gene ontology enrichment analysis of genes regulating mitochondrial function, the tricarboxylic acid cycle, and oxidative stress. We identified the involvement of the mitochondrial inner membrane, oxidoreductase, NADH dehydrogenase, and Ucp3 (Figures S6A and S6B). Expression analysis of select genes involved in metabolic regulation and mitochondrial function in the aorta shows a tendency toward lower expression of *Sdha* and *Pkm2*, higher expression of *Pdhb*, and no effect on *Mdh1* and *Sdhb* (Figure S6C). Moreover, in the aorta of HFD-fed mice treated with an inhibitor of miR-122, the expression of mitochondrial electron transport chain (ETC) complexes was lower at both gene and protein levels (Figures 3C and 3D). In the aortic immunoblot preparation, the mitochondria of aortic smooth muscle cells also contribute to the phenotype. We immunostained the vasculature for Ndufs4, a subunit of complex I (CI), and observed lower levels in vascular endothelial cells, as indicated by vWF (Figure 3E). Next, we determined the oxygen consumption rate (OCR) in permeabilized aorta to assess mitochondrial function. The OCR was higher at basal conditions and during sequential addition of pyruvate/malate (Pyr/Mal) and ADP in the aorta of HFD-fed mice receiving γP-122-I than in those receiving γP-SC (Figure 3F). The increase in OCR despite reduced ETC complex expression was surprising. Therefore, we calculated the coupling efficiency to measure ATP synthesis linked to oxygen consumption. Coupling efficiency was determined by subtracting the ratio of leak-associated oxygen flux to oxphos-associated oxygen flux from 1, as described previously.^26^^,^^27^ Our data indicate that miR-122 inhibition improves CI and CI + CII coupling efficiency (Figure 3G). An increase in OCR and a decrease in ETC complex levels in the aorta of HFD-γP-122-I mice reinforce miR-122’s impact on ETC function in the vasculature.

### miR-122 regulates mitochondrial function in endothelial cells

**Figure 4:**
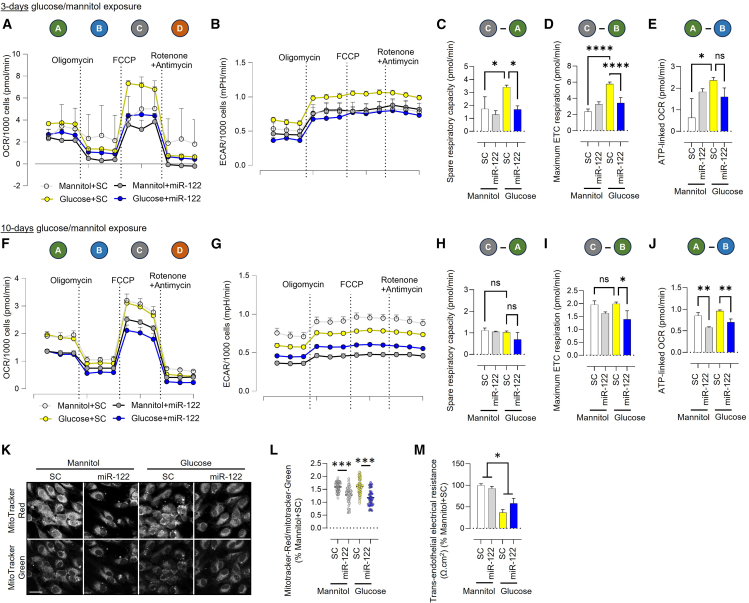
miR-122 overexpression impairs mitochondrial function in HUVECs under hyperglycemic conditions (A–E) Mitochondrial function was assessed using the Seahorse XF Analyzer. Oxygen consumption rate (OCR; A) and extracellular acidification rate (ECAR; B) were measured in HUVECs transfected with a miR-122 mimic or control under basal (5.5 mM glucose + 25 mM mannitol) and hyperglycemic (30.5 mM glucose) conditions for 3 days. OCR was measured at baseline (A), after addition of oligomycin (B), after addition of FCCP (C), and after addition of a mixture of rotenone and antimycin A (D) (*n* = 4–6). Mannitol was used as an osmolarity control. Quantification of spare respiratory capacity (C), maximum ETC respiration capacity (D), and ATP-li nked OCR (E) is shown (*n* = 4–6). The formula above each quantification shows how the parameter was calculated based on the OCR data in “a.” (F–J) OCR (F) and ECAR (G) in HUVECs maintained under basal (5.5 mM glucose + 25 mM mannitol) and hyperglycemic (30.5 mM glucose) conditions for 10 days and transfected with a miR-122 mimic or control. Transfection with the miR-122 mimic or control was performed 3 days prior to the assay. OCR was measured at baseline (A), after addition of oligomycin (B), after addition of FCCP (C), and after addition of a mixture of rotenone and antimycin A (D) (*n* = 3–5). Quantification of spare respiratory capacity (H), maximum ETC respiration capacity (I), and ATP-linked OCR (J) is shown (*n* = 3–5). The formula above each quantification shows how the parameter was calculated based on the OCR data in “F.” (K and L) MitoTracker-Red and MitoTracker-Green staining in SC- and miR-122-transfected HUVECs under basal and hyperglycemic conditions (k), and quantification of the MitoTracker-Red to MitoTracker-Green ratio (L) *n* = 50, where “*n*” represents the number of cells quantified per group. Magnification ×63; scale bars, 20 μm. Quantification was performed for each cell derived from five high-magnification images per experimental condition. Data are rrepresentative of three independent experiments. (M) Effect of glucose and miR-122 on *trans*-endothelial electrical resistance in HUVECs (*n* = 4–5). ∗*p* < 0.05, ∗∗*p* < 0.01, ∗∗∗*p* < 0.001, and ∗∗∗∗*p* < 0.0001 *vs.* the indicated group. Data are shown as mean ± S.E.M.

To assess the impact of miR-122 on mitochondrial function in endothelial cells, we conducted Seahorse mito-stress assays to measure OCR and extracellular acidification rate (ECAR) in HUVECs overexpressing miR-122 under both basal and hyperglycemic conditions. Measurements were recorded at baseline and following sequential injections of oligomycin, carbonyl cyanide-4-(trifluoromethoxy)phenylhydrazone (FCCP), and a combination of rotenone and antimycin A. Oligomycin inhibits ATP synthase (Complex V), reducing ATP-linked respiration. FCCP acts as a protonophore, collapsing the mitochondrial membrane potential by allowing protons to flow freely across the inner mitochondrial membrane, thereby uncoupling the ETC from ATP production and driving maximal respiration. Rotenone and antimycin A inhibit CI and CIII, respectively, shutting down mitochondrial respiration. These steps enabled quantification of spare respiratory capacity, maximum ETC respiration, and ATP-linked respiration. A higher level of glucose increased spare respiratory capacity, maximal ETC respiration, and ATP-linked OCR in HUVECs, which was inhibited by the overexpression of miR-122 (Figures 4A–4E). Multi-passage exposure of HUVECs to hyperglycemia inhibits the hyperglycemia-induced increase in spare respiratory capacity and maximal ETC respiration, but miR-122 overexpression decreases maximal ETC respiration and ATP-linked OCR under hyperglycemic conditions (Figures 4F–4J). These data suggest that miR-122 impairs mitochondrial adaptability. Staining HUVECs with membrane potential-dependent MitoTracker Red and membrane potential-independent MitoTracker Green showed that miR-122 reduces the number of mitochondria with high membrane potential under both basal and hyperglycemic conditions (Figures 4K and 4L). We also measured *trans*-endothelial electrical resistance to assess the impact of miR-122 on endothelial barrier integrity. We found that while hyperglycemia reduced *trans*-endothelial electrical resistance, miR-122 overexpression had no significant effect under either basal or hyperglycemic conditions (Figure 4M).

## Discussion

miR-122 is highly expressed in the liver and regulates lipid metabolism and macrophage activation.^28^^,^^29^ Typically miRs are secreted as AGO2:miR complexes, loaded into extracellular vesicles, and HDL:miR complexes, or passively released during cell membrane disruption.^30^^,^^31^ Hepatocytes actively export miR-122 into the circulation through vesicular and non-vesicular mechanisms, and the health condition such as obesity and diabetes aggravate this process.^3^^,^^32^^,^^33^^,^^34^ Although circulating miR-122 is widely used as a biomarker of liver injury, increasing evidence suggests that its release is a regulated process and could signal to distant organs and tissues.^35^ Systemic miR-122 inhibition improves glycemic control and vascular health,^9^^,^^10^^,^^11^ but the underlying mechanism remains unknown. Our study demonstrates that miR-122 impairs mitochondrial function in vascular endothelial cells, and its systemic inhibition using γPNA technology improves endothelial function in prediabetic mice.

Systemic miR-122 inhibition enhanced endothelial function and glucose tolerance in HFD-fed mice.^9^ It remains unknown whether the improvement in endothelial function following miR-122 inhibition is due to decreased miR-122 effects in endothelial cells or is secondary to improved glycemic control. The present study shows that the endothelial cell-targeted miR-122 inhibitor exhibits similar efficacy in restoring endothelial vasorelaxation while showing milder effects on miR-122 expression in non-target tissues (e.g., pancreas and kidney) and on whole-body glucose disposal during the glucose tolerance test. Our selection of a VCAM-1 targeting peptide for endothelial cell targeting is based on single-cell transcriptomics, which reveals a high level of VCAM-1 in endothelial cells^19^ and an increase in its expression in endothelial cells of diabetic mice.^36^^,^^37^ Previous reports also demonstrate the suitability of VHPK for endothelial cell targeting.^38^ We recognize that targeting endothelial cells using VHPK will also target non-endothelial cells expressing VCAM-1, such as proximal tubular cells and Kupffer cells. As e-γP-122-I improved endothelial function without enhancing glucose disposal compared to non-targeted γP-122-I, broader miR-122 inhibition might be necessary for metabolic benefits. We noted that γP-122-I had a similar effect on miR-122 in the aorta, kidney, and liver, whereas the VCAM-1-targeting e- in the expression of VCAM-1 between endothelial cells of different origins^39^ and γP-122-I had a distinct impact on the kidney (Figure S2E). Differences in the abundance of endothelial cells in a specific tissue could contribute to such differences in miR-122 inhibition in the aorta and kidney in response to e-γP-122-I. In addition, our results show that endothelial cells internalize miR-122 through an NRP-1-dependent mechanism, as evidenced by reduced miR-122 internalization upon neutralizing the NRP-1 b1b2 domain. Previous studies have shown that the cell surface receptor NRP-1 facilitates the internalization of AGO2/miRNA complexes.^40^ These data show that γP-122-I improves endothelial function and whole-body glycemic control in HFD-fed mice, and e-γP-122-I offers no competitive advantage over γP-122-I.

In transcriptomics analysis of the mouse aorta, we also observed deregulation of mitochondrial genes. Previous studies have shown the role of miR-122 in mitochondrial function in hepatocytes, cardiomyocytes, and pancreatic acinar cells.^41^^,^^42^^,^^43^ Our results show the role of miR-122 in regulating mitochondrial function in vascular endothelial cells. Specifically, miR-122 inhibition improved mitochondrial OCR in the aorta of HFD-fed mice, despite a decline in the expression of mitochondrial ETC complexes, indicating enhanced mitochondrial activity. *In vitro*, miR-122 overexpression reduced the spare respiratory capacity under hyperglycemic conditions, along with the proportion of mitochondria exhibiting high membrane potential, a population generally associated with more efficient ATP production. Previous studies have reported a decrease in spare respiratory capacity under hyperglycemia.^44^^,^^45^^,^^46^ In contrast, other reports suggest that senescent HUVECs, often induced by hyperglycemic stress, exhibit higher spare respiratory capacity than their younger counterparts.^47^ Our data show that short-term hyperglycemic exposure increases both spare and maximal ETC respiration, whereas prolonged hyperglycemia does not elicit this compensatory enhancement. Importantly, in both settings, miR-122 overexpression consistently suppressed mitochondrial respiratory capacity. Although the impact of sustained miR-122 elevation on mitochondrial function under chronic hyperglycemia merits further investigation, our findings indicate that miR-122 inhibition may preserve mitochondrial energetics and improve cellular efficiency within the vasculature. The dual role of miR-122 in the vasculature and glycemic control positions it as a promising therapeutic for diabetes and its complications. Elevated circulating miR-122 in conditions such as obesity and diabetes contribute to endothelial apoptosis, atherosclerosis, and impaired insulin secretion.^1^^,^^6^^,^^9^^,^^10^^,^^11^

Systemic and cell-specific miR-122 inhibition strategies offer potential therapeutic avenues but require careful consideration of tissue-specific effects and safety. The γ-position modification promotes the formation of right-handed helical structures that confer stronger binding to RNA targets.^48^^,^^49^ We previously showed that γP-122-I forms a heteroduplex with miR-122 with very high affinity.^9^ This modification addresses another common challenge to nucleic acid-based therapeutics; undesired immune system activation.^50^^,^^51^ γPNAs are safe in electronic barcoding^52^ and gene editing/targeting.^51^^,^^53^^,^^54^^,^^55^^,^^56^^,^^57^ Supporting the translational potential of γP-122-Is, OLP-1002 (another PNA) is undergoing a phase 2 clinical trial, and phase 1 concluded with no safety concerns.^58^ Although multiple independent studies have demonstrated that PNAs and γPNAs are well tolerated *in vivo*—including intravenous γPNA delivery without detectable toxicity,^59^ repeated high-dose systemic PNA administration in *mdx* mice,^60^ and favorable safety profiles for therapeutic PNAs targeting N-myc,^61^^,^^62^ c-myc,^63^ and multiple oncomiRs^64^—the long-term toxicological effects of chronic γPNA dosing remain unknown. Short-term tolerability observed with γP-122-I in the present study is consistent with prior work, including hepatocyte- and kidney-targeted PNA delivery without detectable toxicity,^65^^,^^66^ and investigational new drug (IND)-enabling γPNA programs demonstrating safety in mice and non-human primates.^67^^,^^68^ However, comprehensive long-term studies assessing immunogenicity, biodistribution, and multi-dose pharmacology will be essential for fully defining the chronic safety profile of γPNA-based therapeutics. Although long-term safety is essential for eventual clinical translation, the goal of the current study was to establish proof-of-concept efficacy and short-term tolerability of γP-122-I within a well-defined experimental window.

While our study provides novel insights into the role of miR-122 in endothelial cells, several questions remain unanswered. For example, whether NRP-1 is the exclusive receptor for miR-122 entry into the endothelial cells, precise molecular mechanisms by which miR-122 regulates mitochondrial function, and alternative mechanisms through which miR-122 could impact endothelial cell function, such as regulation of miR-204 expression^23^^,^^69^ and TLR-8^70^ activation by miR-122. In addition, the long-term effects of miR-122 inhibition on systemic metabolism and vascular health warrant further investigation.

Despite the therapeutic potential of miR inhibition, its clinical adoption has been limited by concerns over off-target effects and the disappointing outcomes of past trials.^71^^,^^72^^,^^73^ However, the ability of miRs to modulate multiple genes simultaneously may offer a unique advantage, reflecting the natural complexity of physiological gene regulation shaped by endogenous miRs. miR-122 inhibitors have been tested for hepatitis C treatment in clinical trials but have faced setbacks due to adverse reactions. Advances in chemical engineering, such as the use of γPNA-based inhibitors, may overcome these limitations by enhancing target specificity and minimizing toxicity. We demonstrate that miR-122 enters endothelial cells and affects mitochondrial function. The contribution of our study lies in addressing some challenges in miR therapeutics and demonstrating that γPNA technology effectively improves glycemic control and endothelial function. These findings highlight miR-122 as a promising therapeutic target and lay the foundation for developing safer and more effective therapies for diabetic vasculopathy.

## Materials and methods

### Design and synthesis of γP-SC, γP-122-I, e-γP-SC, and e-γP-122-I

The γP-SC and γP-122-I were synthesized as described.^9^ A detailed description is provided in the supplemental information.

### General experimental

All animal experiments were approved by the Institutional Animal Care and Use Committee (IACUC) of the University of Iowa and were conducted in accordance with the National Institute of Health (NIH) Guide for the Care and Use of Laboratory Animals. Mice aged 8 to 16 weeks, housed in a conventional pathogen-free animal facility on a 12-h dark/light cycle and with *ad libitum* access to rodent chow and drinking water, were used for all experiments. Starting at 8 weeks of age, mice were fed a HFD (TD88137, Envigo, IN, USA; containing 21.2% wt/wt fat, 48.5% wt/wt carbohydrate, 17.3% wt/wt protein, and 0.2% wt/wt cholesterol) for 8 weeks. Two weeks after dietary intervention, these mice were intraperitoneally injected with γP-SC, γP-122-I, e-γP-SC, or e-γP-122-I (0.25 μmol.kg^−1^) for 6 weeks. Age-matched mice fed a ND served as the control group. All compounds tested *in vivo* were >95% pure by HPLC. Body weight and blood glucose levels were measured every 2 weeks in ND, HFD-saline, HFD-γP-SC, and HFD-γP-122-I mice. Fasting blood glucose levels were measured in mice that had been fasted for 6 h. For intraperitoneal glucose tolerance test (IPGTT), after 6 h of fasting, mice were injected intraperitoneally with glucose at a dose of 2 g.kg^−1^, and glucose levels were measured at various time points. Adiposity was calculated as the combined weight of white adipose tissue (epididymal, WAT) and brown adipose tissue (interscapular, BAT) per 100 g body weight. The e-γP-SC and e-γP-122-I were administered to 8-week-old db/+ or *db/db* mice for 6 weeks at a dose of 0.25 μmol.kg^−1^ daily.

### Vascular reactivity

Vascular reactivity was determined as previously described.^74^ Briefly, the aortic rings (1.5–2.0 mm wide) were placed in ice-cold, oxygenated Krebs-Ringer bicarbonate (KB) solution. The rings were placed in an oxygenated organ bath filled with KB solution, and the organ baths were maintained at 37°C. Each ring was suspended in a wire myograph system (DMT Instruments, FL, USA). The extent of endothelium-dependent vascular relaxation was determined by generating dose-response curves to acetylcholine (ACh,10^−9^-10^−5^ M) on aortic rings that had been precontracted wih phenylephrine (PE, 10^−6^ M). Endothelium-independent vasorelaxation was determined by generating dose-response curves to SNP on aortic rings precontracted with PE (10^−6^ M). Vascular relaxation elicited by ACh and SNP was represented as a percentage of relaxation. Aortic rings that did not react to potassium chloride (KCl) or demonstrated auto-relaxation were eliminated.

### Cell culture

HUVECs (Cat. No. CC-2519) were procured from Lonza (Mapleton, IL, USA) and cultured in endothelial cell growth medium supplemented with growth factors (PromoCell; C-22211 and C-39211, Heidelberg, Germany). To study the role of NRP-1 in miR-122 uptake in endothelial cells, HUVECs were treated with 2% serum from either ND- or HFD-fed mice in the presence of NRP-1 (Sigma MABS2299) or IgG antibody (2 ug/mL). Further, HUVECs were exposed for 24 h to 2% serum derived from FAM-miR-122-treated HFD-fed mice, followed by immunostaining with anti-NRP-1 (Sigma, MABS2299) and visualization of FAM–miR-122 and NRP-1 using a super-resolution microscope (MI-SIM, CSR Biotech Co., Ltd. Guangzhou, China). To assess the effect of miR-122 on mitochondria with high membrane potential, the HUVECs were transfected with either a SC or miR-122 mimic (miR-122-M) under basal (5.5 mM glucose + 25 mM mannitol) or high-glucose conditions (30.5 mM). Mannitol was used to match osmolarity.^75^^,^^76^^,^^77^ The cells were incubated with MitoTracker Red CMXRos (200 nM; M7512, Invitrogen) and MitoTracker Green FM (200 nM; M7514, Invitrogen). OCR and ECAR were measured using the Seahorse XF96 Analyzer (Agilent) following the MitoStress Test protocol. Briefly, HUVECs were seeded in 60 mm plates and transfected with SC or miR-122 M (20 nM) under basal or high-glucose conditions. Readings were collected either 3 days or 10 days after exposure to the high-glucose/mannitol concentration. After 24 h, the cells were seeded at 40,000 cells/well in XF24 cell culture microplates (Agilent Technologies) and allowed to adhere for an additional 24 h. On the assay day, cells were washed with Seahorse XF assay medium (pH 7.4, containing 2 mM glutamine, 1 mM sodium pyruvate, and 25 mM glucose) and incubated with the assay medium for 1 h at 37°C in a non-CO2 incubator. Following incubation, sequential injections of oligomycin (1 μM), FCCP (1.5 μM), and rotenone/antimycin A (0.5–1 μM each) were performed. Three repeated OCR and ECAR measurements were obtained at baseline and after each substrate injection. Parameters such as ATP-linked respiration, proton leak, coupling efficiency, and spare respiratory capacity were calculated. For measuring endothelial barrier integrity, HUVECs were seeded at 1 × 10^5^ cells/cm^2^ on gelatin-coated transwell inserts (Corning 6.5 mm Transwell with 0.4 μm pore polyester membrane insert, 3470) and treated with SC or miR-122 M under basal or hyperglycemic conditions. *Trans*-endothelial electrical resistance was measured using an epithelial voltohmmeter (EVOM) manual meter (World Precision Instruments, Sarasota, FL). Resistance values were corrected for blank inserts and normalized to membrane area (Ω·cm^2^).

### Transcriptomic analysis

A detailed description is provided in the supplemental information.

### Oxygen consumption by the aortic tissues

Mitochondrial respiration was assessed in aortic tissues using the high-resolution Oxygraph-2K system (Oroboros Instruments, Innsbruck, Austria), as described.^78^ Briefly, excised aortic tissues were cut to expose the endothelial layer and immersed in ice-cold Buffer X (7.2 mM K_2_EGTA, 2.8 mM CaK_2_ EGTA, 20 mM Imidazole, 0.5 mM DTT, 20 mM taurine, 5.7 mM ATP, 14.3 mM phosphocreatine, 6.6 mM MgCl_2_·6H_2_O, 50 mM MES). Approximately 2.0 mg of tissue was incubated in buffer X containing 10 μg/mL saponin for 30 min at 4°C on a rotating shaker to permeabilize the tissue. After permeabilization, the aortic tissues were washed and incubated for 15 min at 4°C in buffer Z (105 mM K-MES, 30 mM KCl, 10 mM KH_2_PO_4_, 5 mM MgCl_2_·6H_2_O, 2.5 mg/mL BSA, and 1 mM EGTA) supplemented with 50 μM pyruvate and 20 μM malate. Mitochondrial respiration was measured in O2K chambers containing 2.5 mL of buffer Z. Oxygen consumption was assessed in response to pyruvate (2 mM) and malate (2 mM), followed by the sequential addition of ADP (1 mM) and succinate (6 mM). To evaluate complex-specific respiration, rotenone (10 μM) was used to inhibit CI, and oligomycin (10 μM) was added to inhibit ATP synthase (complex V). Data acquisition and analysis were performed using DatLab software version 4.3 (Oroboros Instruments). Coupling efficiency was calculated to assess the proportion of respiration linked to ATP synthesis relative to proton leak. Oxygen fluxes were corrected for residual non-mitochondrial respiration measured after antimycin A. For CI–linked respiration, leak respiration was measured with Pyr/Mal in the absence of ADP (CI-L), and OXPHOS respiration was obtained after ADP addition (CI-O). Coupling efficiency for CI was expressed as 1−(CI-L/CI-O). For CII–linked respiration, succinate was added in the presence of rotenone and ADP to obtain OXPHOS respiration (CII-O). At the same time, oligomycin was used to determine leak respiration under the same substrate condition (CII-L). Coupling efficiency for CII was calculated as 1−(CII-L/CII-O). These indices are standard in high-resolution respirometry and reflect the fraction of substrate-driven electron transport that is effectively coupled to ATP synthesis.^27^

### Immunoblotting

A detailed description is provided in the supplemental information.

### Histology and immunohistochemistry

A detailed description is provided in the supplemental information.

### qPCR

RNA was isolated using Trizol. miRs and RNAs were converted to cDNA using the qScript microRNA cDNA Synthesis Kit (Quanta Bio, MA, USA). qPCR for miR-122, miR-29b, miR-148a, miR-133a, *Cpeb1*, *Tgfb1i1*, *Bnc2*, *Ddx60*, *Lama2*, *Pdhb*, *Sdhb*, *Mdh1*, and *Pkm2* was performed using the SYBR Green RT-qPCR Kit, and 18S rRNA was used as an internal control. Serum miR levels were quantified using a constant amount of serum (200 μL). The primer sequences are provided in Table S4.

### Statistical analysis

Statistical analysis was performed using GraphPad Prism (version 8.0). One-way analysis of variance (ANOVA) was used for multiple comparisons, and Tukey’s test was used for post-hoc analysis. An independent sample *t* test was used to determine the significance of differences between the two groups. Nonlinear regression was used to assess the significance of differences between vascular relaxation curves. Briefly, log(agonist) vs. response (three parameters) was used to estimate best-fit parameters such as logEC50, degrees of freedom, and sum of squares (separate and shared). Differences between curves were evaluated using an extra sum-of-squares *F* test. Data are presented as the mean, with error bars representing the standard error of the mean. Results were considered significant where *p* < 0.05.

## Data availability

All data supporting the findings of this study are available within the article and the supplemental information. The raw sequencing data are available in the NCBI Gene Expression Omnibus (GEO): GSE304654. Additional datasets are available from the corresponding authors upon reasonable request.

## Acknowledgments

We acknowledge the Central Microscopy and Research Facility (CMRF) at the University of Iowa, Iowa City, IA. We recognize Dr. Juan E. Abrahante, University of Minnesota Genomics Center (UMGC), MN, for assistance in submitting the transcriptomics data to the NCBI GEO database. We thank CSR Biotech (Guangzhou) Co., Ltd. for live-cell imaging using their commercial super-resolution microscope (MI-SIM), data acquisition, SR image reconstruction, analysis, and discussion. The graphic abstract was created with BioRender.com. This work was supported by grants from the FOEDRC Bridge-to-the-Cure fund and the University of Iowa Start-up fund to Ajit Vikram, a Career Development Award from the 10.13039/100000968American Heart Association-23CDA1037711 to R.R.G., and a UConn Spark Grant to R.B. Ajit Vikram. was partly supported by 10.13039/100000002NIH-R01HL167773, AHA-23CDA1037711, and the FOEDRC Bridge-to-the-Cure fund. R.R.G. was partly supported by the AHA postdoctoral award (828081) and Career Development Grant (23CDA1037711). R01HL158546 partly supported ND.

## Author contributions

R.R.G., V.S.A., and P.D. performed animal and cell culture studies. S.D. helped maintain the animal colony and performed histological analysis. M.P. and V.K. synthesized γP-SC, γP-122-I, e-γP-SC, and e-γP-122-I and performed quality control analysis. Anamika Vikram, H.K., and Ajit Vikram performed the bioinformatics analysis. Ajit Vikram, N.D., A.K.M., V.L., and R.B. designed the research and analyzed the data. R.R.G. and Ajit Vikram prepared the first draft of the manuscript. Ajit Vikram and R.B. secured funding for this work and supervised the project’s progress. All authors have approved the final version of the manuscript.

## Declaration of interests

Ajit Vikram, R.B., and R.R.G. are named as the inventors of U.S. patent 20230322862A1, held by the University of Iowa Research Foundation (UIRF).

## Supplemental information

Data S1. Tables S1 and S2

Document S2. Article plus supplemental information
