# Supplementary material for: Engineered miR-122 inhibitors preserve endothelial mitochondrial function and prevent vascular dysfunction in obesity-associated prediabetes
Source: Mol Ther Nucleic Acids. 2026 Jan 9;37(1):102830. doi: 10.1016/j.omtn.2026.102830 (PMC12860614; doi:10.1016/j.omtn.2026.102830)
Supplement: Document S1. Figures S1–S6 and Tables S3 and S4 [file mmc1.pdf]

OMTN, Volume *37*

Supplemental information

Engineered miR-122 inhibitors preserve endothelial mitochondrial function and prevent vascular dysfunction in obesity-associated prediabetes

Ravinder Reddy Gaddam, Mounika Pathuri, Paroma Deb, Subhash Dwivedi, Anamika Vikram, Vishal Kasina, Veda S. Amalkar, Vitor Lira, Harpreet Kaur, Nirav Dhanesha, Ashutosh Kumar Mangalam, Raman Bahal, and Ajit Vikram

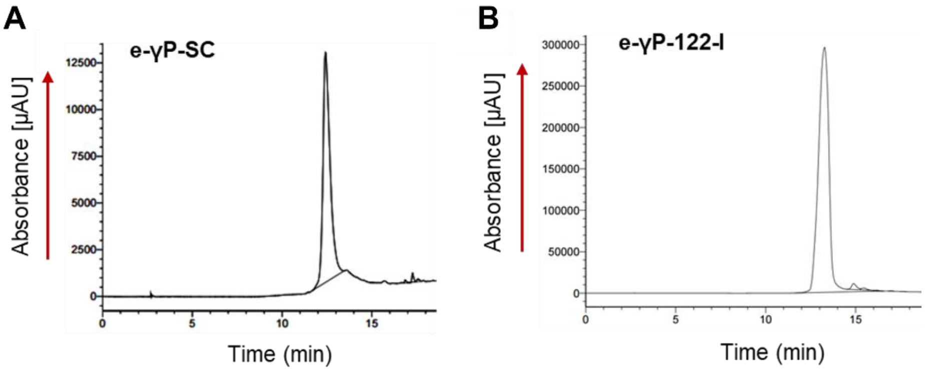

C Table comparing calculated and observed molecular weight of e-γP-SC and e-γP-122-I using MALDI-TOF

PNA	Calculated MW (Da)	Observed MW (Da)
e-γP-SC	10431	10480
e-γP-122-I	10431	10446

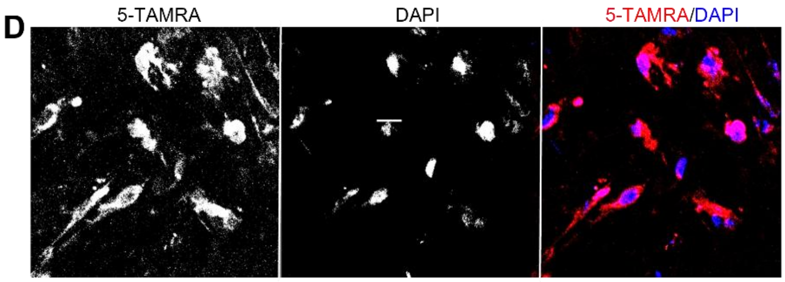

Fig. S1. Characterization of e-γP-122-I. A & B) High-performance liquid chromatography (HPLC) chromatograms of e-γP-SC and e-γP122-I. **C**) Molecular weights of e-γP-SC and e-γP122-I measured by using Matrix-Assisted Laser Desorption/Ionization (MALDI) spectrometry. **D**) Confocal imaging of the aortic endothelial layer following administration of the e-γP122-I-TAMRA (×63).

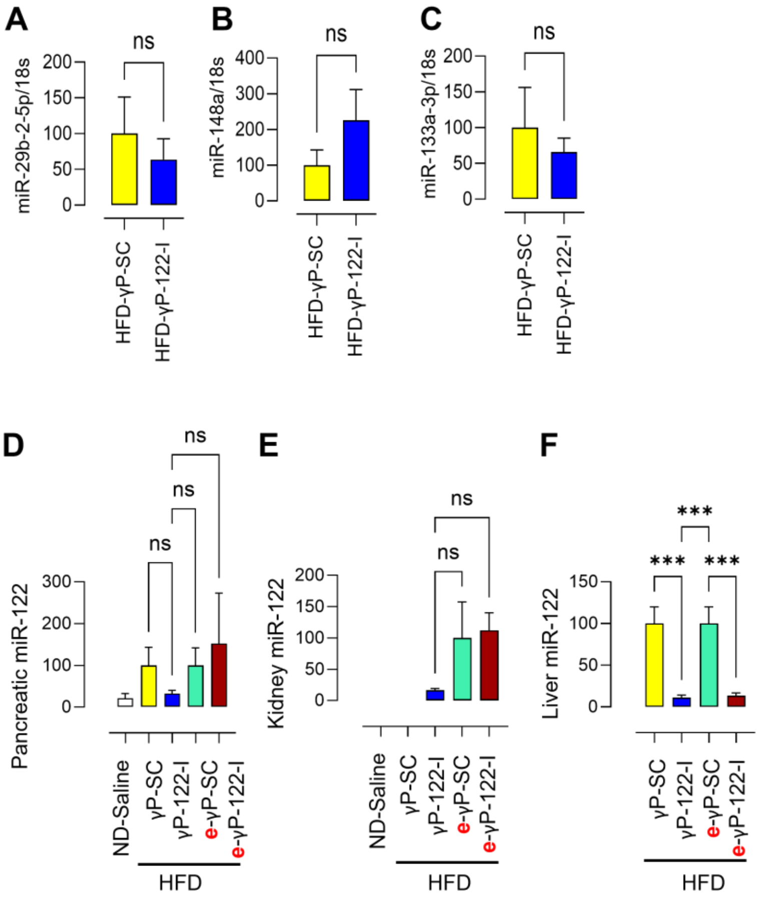

Fig. S2. Effect of γP-122-I on the expression of other miRs in aorta and effect of γP-122-I and e-γP-122-I on expression of miR-122 in pancreas, kidney, and liver. A-C) Effect of γP-SC and γP-122-I on the expression of miR-29b, miR-148a, and miR-133a in the aorta of HFD-fed mice. n = 4. **D-F**) Effect of γP-122-I and e-γP-122-I on pancreatic, kidney, and liver miR-122 levels in normal diet-fed mice receiving saline (ND-Saline), HFD-fed mice receiving γP-SC, γP-122-I, e-γP-SC, or e-γP-122-I. n = 3-6. $^{ns}p > 0.05$, and $^{***}p < 0.001$ vs. the indicated group. Data are shown as mean ± S.E.M.

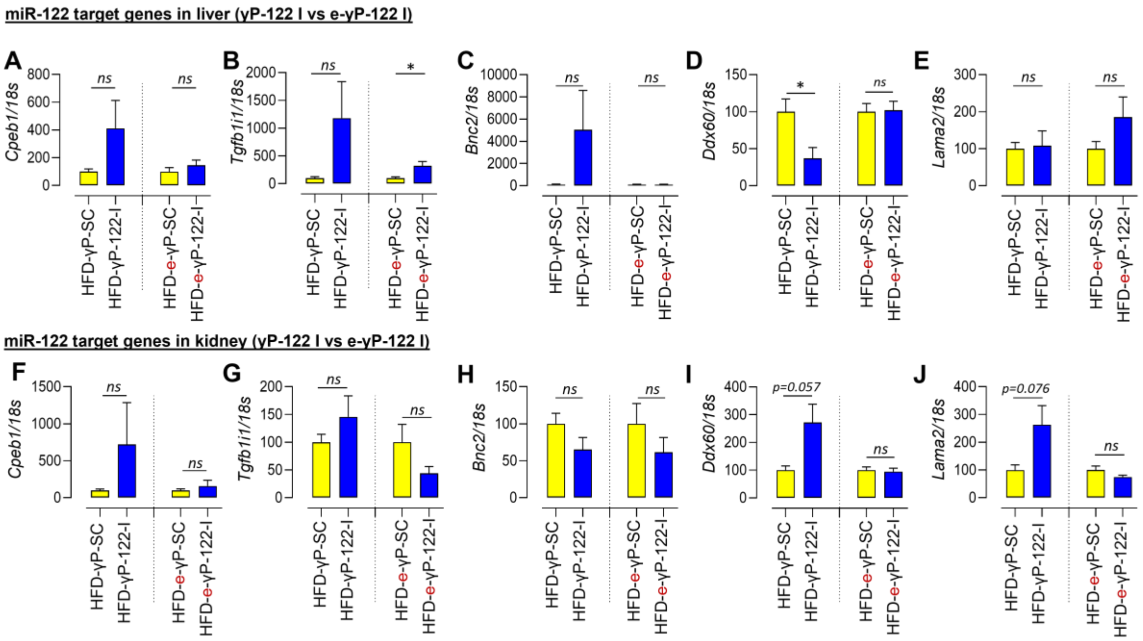

Fig. S3. Effect of γP-122-I and e-γP-122-I on the expression of miR-122 target genes in the liver (A-E) and kidney (F-J). n = 5-6. $^{ns}p > 0.05$ and $^{*}p < 0.05$ vs. the indicated group. Data are shown as mean ± S.E.M.

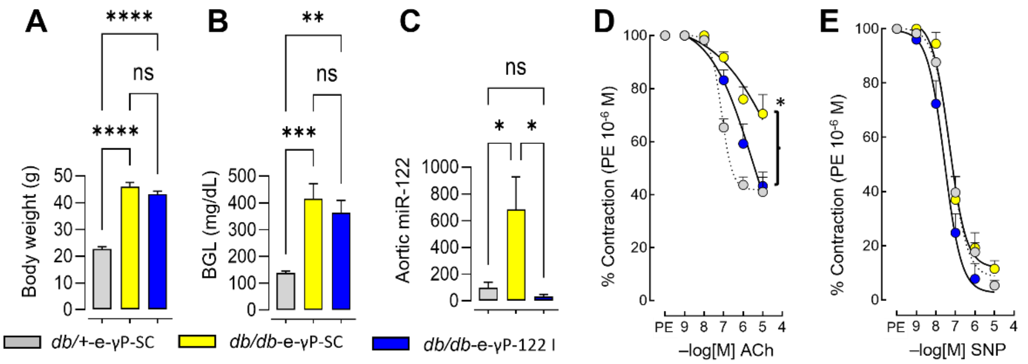

Fig. S4. Vascular effects of e-γP-122-I in *db/db* mice. A-C) Body weight (A), blood glucose level (BGL, B), and aortic miR-122 levels (C) of *db/+* and *db/db* mice receiving e-γP-SC and *db/db* mice receiving e-γP-122-I. Body weight and BGL data were collected five weeks after e-γP-SC or e-γP-122 I treatment whereas aortic miR-122 levels were measured six-weeks after the treatment. n = 6-10. These mice received oligonucleotides at 0.25 µmol kg^{-1} for six weeks and body weight and BGL was measured in the last week of treatment. **D & E**) e-γP-122-I prevent HFD-triggered endothelial dysfunction in the aorta (D) but did not affect the SNP-mediated relaxation (E) in the aorta of *db/db* mice. n = 3-5. Nonlinear regression was used to assess the significant difference between the two vascular relaxation curves. $^{ns}p > 0.05$, *$p < 0.05$, **$p < 0.01$, ***$p < 0.001$, and ****$p < 0.0001$ vs. the indicated group. Data are shown as mean ± S.E.M.

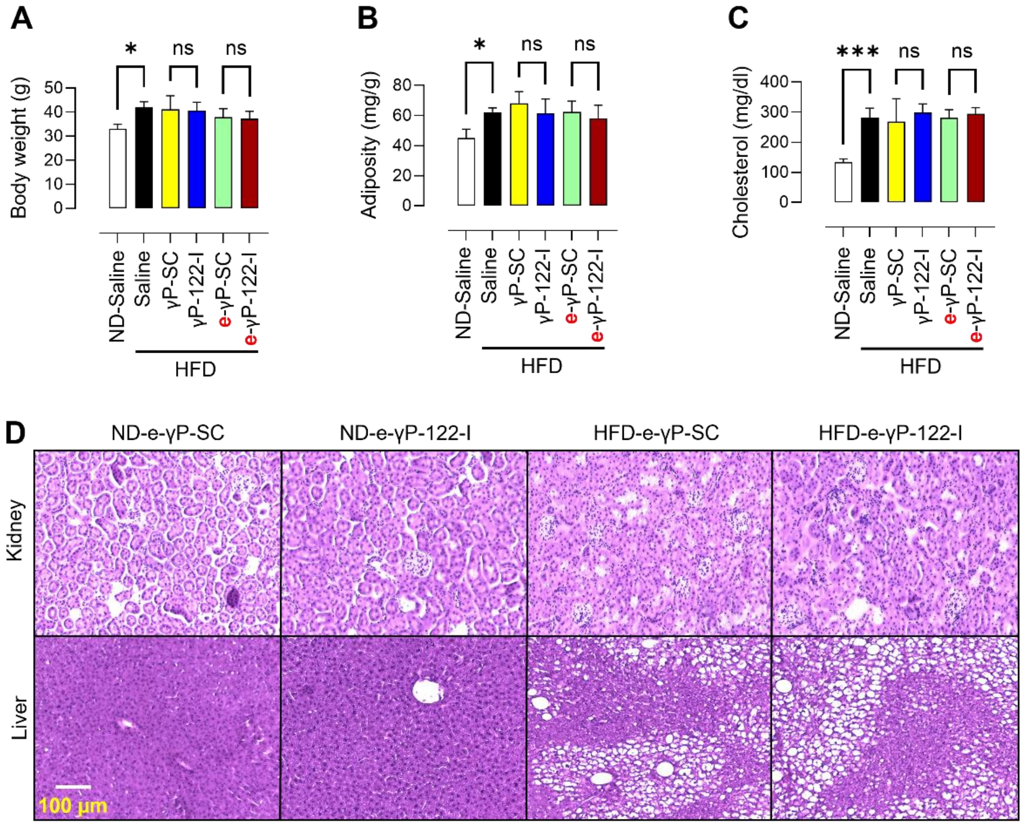

Fig. S5. A-C) Effects of γP-122-I and e-γP-122-I on body weight (A; n = 4-21), adiposity (B; n = 5-8), and cholesterol levels (C; n = 5-6) in normal diet-fed (ND) or HFD-fed mice. The respective controls received either γP-SC or e-γP-SC. **D**) Representative images showing the effects of e-γP-SC and e-γP-122-I on the histology of liver and kidney. ×10. $^{ns}p > 0.05$, $^{*}p < 0.05$, and $^{***}p < 0.001$ *vs.* the indicated group. Data are shown as mean ± S.E.M.

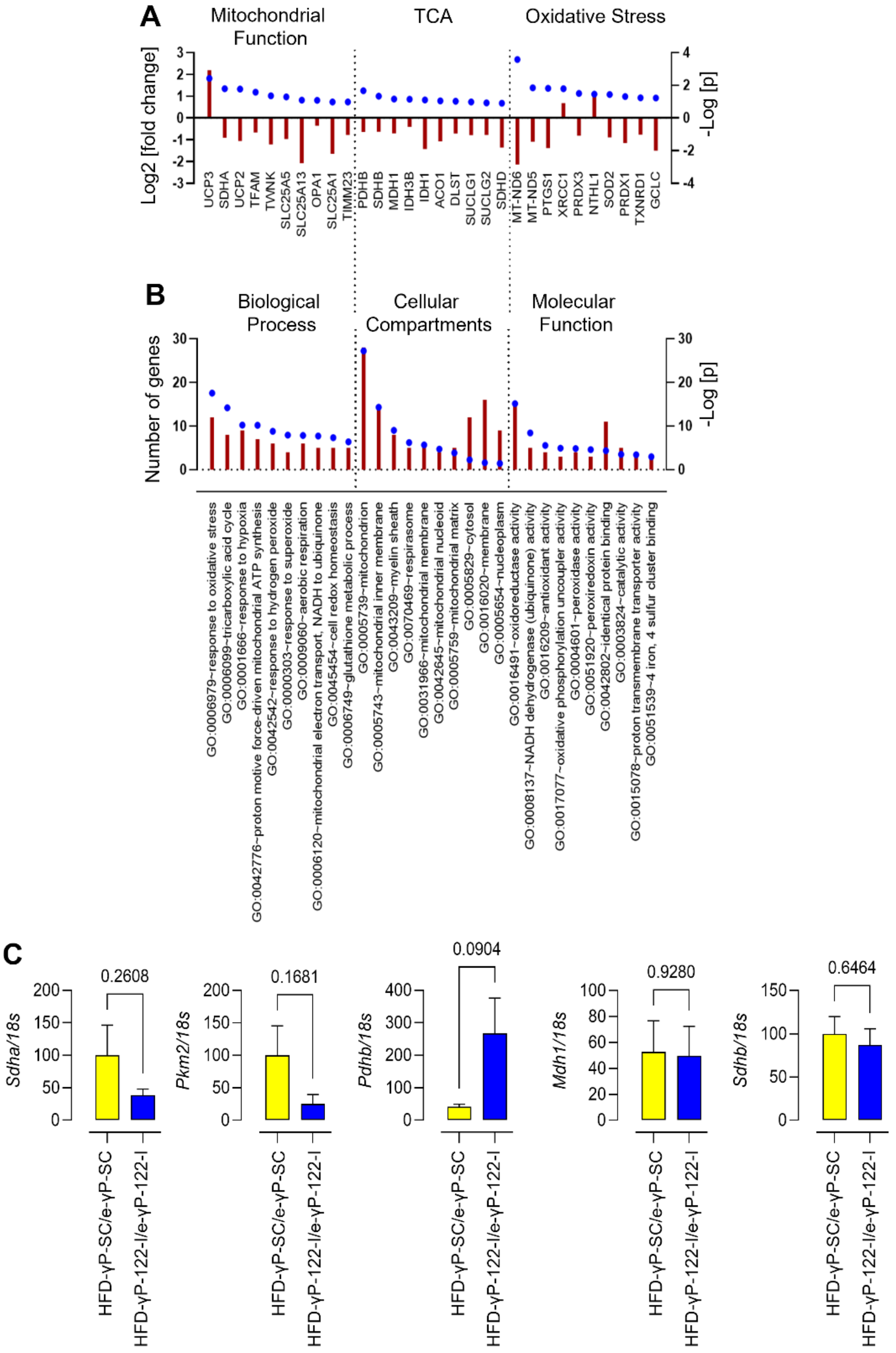

Fig. S6. Pathway analysis. A) Top 10 altered genes related to mitochondrial function, tricarboxylic acid (TCA) cycle, and oxidative stress in the aorta of the HFD-fed mice receiving γP-122-I compared to those receiving γP-SC. The red bar indicates fold change in the expression, while the blue dot indicates the significance. **B**) The gene-ontology enrichment analysis using the Database for Annotation, Visualization, and Integrated Discovery (DAVID) v6.8 shows the top 10 biological processes, cellular components, and molecular functions that change with miR-122 inhibition. The red bar indicates the number of genes, and the blue dot shows the statistical significance. **C**) Expression of *sdha, pkm2, pdhb, mdh1,* and *sdhb* in the aorta of the HFD-fed mice receiving miR-122 inhibitor compared to control. p value and comparison are indicated. Data are shown as mean ± S.E.M.

Supplemental methods

Design and synthesis of γP-SC, γP-122-I, e-γP-SC, and e-γP-122-I. The γP-SC and γP-122-I were synthesized, as described.[9] Briefly, BOC (tert-butyloxycarbonyl)-protected diethylene glycol γ monomers were used for γP-122-I, which were procured from ASM Research Chemicals (Hannover, Germany). The monomers were vacuum-dried before the start of solid-phase synthesis. Approximately 100 mg of lysine-loaded resin was soaked in dichloromethane (DCM) for 5 hours in a reaction vessel. DCM was drained, and the resin was deprotected using a mixture of trifluoroacetic acid and m-cresol for 5 minutes. This deprotection step was repeated thrice, then the resin was washed with DCM and N, N-dimethylformamide (DMF). The monomer was dissolved in a coupling solution comprising 0.2M N-Methyl pyrrolidone (NMP), 0.52M Di-isopropylethylamine (DIEA), and 0.39M O-Benzotriazole-N, N, N', N'-tetramethyl-uroniumhexafluoro-phosphate (HBTU). The coupling solution was added to the reaction vessel and rocked for 2 hours. The resin was capped using a capping solution (mixture of NMP, pyridine, and acetic anhydride) and then washed with DCM. The entire process was repeated until the last monomer was added. 5-carboxy tetramethylrhodamine (TAMRA) was conjugated to the N terminus of γP-122 I. γPNA was cleaved from the resin using a cleavage cocktail (thioanisole, m-cresol, TMFSA, TFA (1:1:2:6), and the vessel was rocked for 1.5 hours. The γPNA was then collected and precipitated using diethyl ether, centrifuged at 3500 rpm for 5 minutes, washed twice with diethyl ether, and vacuum dried. HPLC-purified γPNA, and its absorbance was measured by Nanodrop (Thermo-fisher Scientific, MA). The extinction coefficients of the individual monomers used to calculate the PNA concentration are: 6,600 $M^{-1}cm^{-1}$ (C), 13,700 $M^{-1}cm^{-1}$ (A), 8,600 $M^{-1}cm^{-1}$ (T), and 11,700 $M^{-1}cm^{-1}$ (G). VHPK conjugation to the γP-SC and γP-122-I to generate e-γP-SC and e-γP-122-I.

Transcriptomic analysis: RNA was isolated using Trizol, quantified by the RiboGreen assay, and RNA integrity was assessed using capillary electrophoresis (Agilent BioAnalyzer 2100). RNA samples were used to generate Illumina sequencing libraries using Illumina's TruSeq RNA Sample Preparation Kit or Stranded mRNA Sample Preparation Kit. The libraries were amplified, and the final library size distribution was validated using capillary electrophoresis. The library size was quantified using both fluorimetry (PicoGreen) and quantitative PCR (Q-PCR). Indexed libraries are then normalized, pooled, and size-selected to 320 bp using Pippin HT. Pooled libraries are denatured and diluted to the appropriate concentration for clustering. The libraries are then loaded onto the NovaSeq paired-end flow cell. Upon completion of reading 1, a 7-base pair index read is performed in the case of single-indexed libraries. The clustered library fragments were synthesized in the reverse direction, thus producing the template for paired end read 2. Illumina Real-Time Analysis software generates base call files for each sequencing cycle. The base call files and run folders were streamed to servers and maintained at the Minnesota Supercomputing Institute. Primary analysis and demultiplexing were performed using Illumina's bcl2fastq v2.20.

Immunoblotting: Protein samples were resolved on 4-15% SDS-PAGE and transferred to nitrocellulose membranes. Antigen-primary antibody complexes were incubated with horseradish-peroxidase (HRP)-conjugated secondary antibodies and visualized using a western blotting luminol reagent (Thermofisher Scientific USA). Anti-Oxphos (Thermofisher Scientific, mAb #45-8199) and anti-Gapdh (Thermofisher Scientific, MA5-33140) were used at a working dilution of 1:1000. Images were captured and quantified using Image Lab (BioRad, USA) software, and intensity values were normalized to Gapdh.

Histology and immunohistochemistry: Formalin-fixed paraffin-embedded tissue (kidney and liver) sections (5 µm) were stained using hematoxylin and eosin, and images were captured using the Rebel microscope (Echo, California, USA). The immunostaining of aortic sections was performed as previously described.[61] The anti-vWF (Abcam, pAb #ab11713) and anti-NDUFS4 (Thermofisher Scientific, PA5-98004) antibodies were used at a working dilution of 1:100. The

images were captured using a Zeiss confocal microscope (Model LSM 710) or commercial super-resolution microscope (MI-SIM, CSR Biotech Co., Ltd. Guangzhou, China).

Excel spreadsheets

Table S1: IPA Analysis of Genes involved in Disease and Function.

Supplied as an Excel file

Table S2: IPA Analysis of Genes involved in Signaling Pathways

Supplied as an Excel file

Table S3: Gene Ontology Analysis of Top 10 Genes

	Term	Genes	p	
Biological ¦	GO:0050853~B cell receptor signaling pathway	5	0.000402	3.395785
	GO:0046485~ether lipid metabolic process	3	0.001271	2.895843
	GO:0009725~response to hormone	5	0.001479	2.830159
	GO:0007169~cell surface receptor protein tyrosine kinase signaling pathway	6	0.00184	2.735068
	GO:0006584~catecholamine metabolic process	3	0.002156	2.666291
	GO:0006915~apoptotic process	14	0.002329	2.632823
	GO:0035556~intracellular signal transduction	11	0.002437	2.613207
	GO:0006629~lipid metabolic process	15	0.002477	2.606085
	GO:0016310~phosphorylation	14	0.003336	2.476754
	GO:0055085~transmembrane transport	11	0.003922	2.406504
Cellular co	GO:0005737~cytoplasm	90	1.13E-05	4.94605
	GO:0016020~membrane	101	1.45E-05	4.839719
	GO:0005886~plasma membrane	77	6.2E-05	4.207442
	GO:0009986~cell surface	18	0.000319	3.495796
	GO:0048471~perinuclear region of cytoplasm	18	0.000528	3.277464
	GO:0009897~external side of plasma membrane	14	0.000896	3.047761
	GO:0005783~endoplasmic reticulum	27	0.001059	2.975297
	GO:0043231~intracellular membrane-bounded organelle	19	0.0021	2.67786
	GO:0005789~endoplasmic reticulum membrane	18	0.005485	2.260855
	GO:0016324~apical plasma membrane	11	0.005926	2.227216
Molecular '	GO:0042802~identical protein binding	34	0.000134	3.872505
	GO:0016491~oxidoreductase activity	16	0.000135	3.868987
	GO:0022857~transmembrane transporter activity	8	0.001148	2.940142
	GO:0016740~transferase activity	28	0.001387	2.858068
	GO:0019899~enzyme binding	12	0.001569	2.80429
	GO:0000166~nucleotide binding	27	0.001905	2.720156
	GO:0016301~kinase activity	14	0.003031	2.518408
	GO:0004672~protein kinase activity	12	0.003206	2.494103
	GO:0003824~catalytic activity	9	0.003273	2.485048
	GO:0004713~protein tyrosine kinase activity	6	0.004248	2.371847

Table S4. The sequence of primers and mature microRNAs

mRNA	Primer Sequence	
	Forward	Reverse
Pkm2 (mouse)	5’-TGC TGC AGT GGG GCC ATT AT-3’	5’-GAG TCA CGG CAA TGA TAG GA-3’
Cpeb1 (mouse)	5'-TTT CAA GCC TTC GCA TTT CCC-3'	5'-GGA CCC AAC GCC CAT CTT TA-3'
Tgfb1i1 (mouse)	5'-AAG GCA GTC TGG ACA CCA T-3'	5'-ACA ACC GCT GCA AAG GAA G-3'
Bnc2 (mouse)	5'-GCT GCA CTT GAC AAC CAG CAT-3'	5'-ATG TTT ACA CTG ATC ACA CGT CC-3'
Ddx60 (mouse)	5'-AAG TGA TGA GCC TTT GTT GAG G-3'	5'-CTC CCA CAT TCA AAT CCA GGC-3'
Lama2 (mouse)	5'-GAC AGC GTG GCC AAA ACG AA-3'	5'-AGT GCC TGC ATC TGC AAT GAT-3'
Sdha (mouse)	5'-GAG ATA CGC ACC TGT TGC CAA G-3'	5'-GGT AGA CGT GAT CTT TCT CAG GG-3'
Pdhb (mouse)	5'-AGG AGG GAA TTG AAT GTG AGG T-3'	5'-ACT GGC TTC TAT GGC TTC GAT-3'
Sdhb (mouse)	5'-AAG AAG GAT GAG TCC CAG GAG-3'	5'-CTT GTC TCC GTT CCA CCA GTA-3'
Mdh1 (mouse)	5'-ATG ATG GGT GTT CTG GA G-3'	5'-TCA CAT TGG CTT TCA GTA GG-3'
PKM2 (human)	5'-ATC GTC CTC ACC AAG TCT GG-3'	5'-GAA GAT GCC ACG GTA CAG GT-3'
18s (mouse and human)	5’-GCC GCT AGA GGT GAA ATT CTT A-3’	5’-CTT TCG CTC TGG TCC GTC TT-3’
**microRNA**	**miR Sequence**	**Primer Sequence**
miR-122-5p	5'-UGG AGU GUG ACA AUG GUG UUU G-3'	5’-TGG AGT GTG ACA ATG GTG TTT G-3’
miR-29b-2-5p	5’-CUG GUU UCA CAU GGU GGC UUA G-3'	5’-TGG TTT CAC ATG GTG GCT TA-3’
miR-148a-3p	5'-UCA GUG CAC UAC AGA ACU UUG U-3'	5’-CGC TCA GTG CAC TAC AGA ACT TT-3’
miR-133a-3p	5'-UUU GGU CCC CUU CAA CCA GCU G-3'	5’-CTT TGG TCC CCT TCA ACC AG-3’
